# A Novel Quantification System Combining iTRAQ Technology and Multi-Omics Assessment to Predict Prognosis and Immunotherapy Efficacy in Colon Cancer

**DOI:** 10.3389/fbioe.2022.862619

**Published:** 2022-04-04

**Authors:** Tianyi Xia, Junnan Guo, Bomiao Zhang, Weinan Xue, Shenhui Deng, Yanlong Liu, Binbin Cui

**Affiliations:** ^1^ Department of Colorectal Surgery, Harbin Medical University Cancer Hospital, Harbin Medial University, Harbin, China; ^2^ Department of Anesthesiology, The Fourth Affiliated Hospital of Harbin Medical University, Harbin, China

**Keywords:** colon cancer, left-sided, right-sided, multi-omics, prognosis, immunotherapy

## Abstract

**Background:** Colon cancer is one of the most common cancer types, although it has certain unique genetic features. This study aimed to develop a unique score for assessing prognosis and immunotherapy efficacy using integrated multi-omics analysis.

**Methods:** Isobaric tagging for relative and absolute quantification (iTRAQ) based proteomic analysis was used to screen differentially expressed proteins (DEP) between tumor and normal samples. DEP mRNA obtained from TCGA were clustered into different categories to show landscape-related prognosis and function. Following that, DEG was extracted from DEP mRNA, and the DEP-related score (DEPRS) was constructed to investigate the difference in immunotherapy prognosis and sensitivity. Finally, WCGNA, random forest, and artificial neural networks were used to screen for key genes. The prognostic value and protein level of these genes were validated.

**Results:** A total of 243 DEPs were identified through iTRAQ analysis, and the corresponding DEP mRNA was clustered into three. Following a series of tests, 1,577 DEGs were identified from overlapped DEP mRNA clusters and were classified into three gene clusters. The two types of clusters described above shared comparable characteristics in terms of prognosis and function. Then, it was established that a high DEPRS indicated a poor prognosis and DEPRS had significant associations with TMB, MSI status, and immunotherapeutic response. Finally, the key genes HART3 and FBLN2 were identified and were found to be implicated in immunotherapy and prognosis.

**Conclusion:** The development of a DEPRS based on multi-omics analysis will aid in improving our understanding of colon cancer and guiding a more effective immunotherapy strategy. DEPRS and key genes are used as biomarkers in the clinical evaluation of patients.

## Introduction

Colorectal cancer (CRC) is the third most common cancer in the world, and it has a significant impact on both the global economy and patients’ lives. Tumor metastasis is the cause of death in half of all patients with CRC (Siegel et al., 2019). Globally, 1,400,000 new cases and 700,000 CRC-related deaths were reported in 2018 (Bray et al., 2018). Colon cancer (CC) accounts for approximately 70% of all CRC (Ahmed, 2020), and CC can be classified into two distinct diseases: left-sided colon cancer (LCC) and right-sided colon cancer (RCC). Due to their unique embryonic origins, LCC and RCC exhibit a variety of clinical characteristics, including drug sensitivity. Additionally, in our previous study, we established the genetic distinction between LCC and RCC in terms of immunotherapy and prognosis (Guo et al., 2021). Based on difference in LCC and RCC, we want to develop a novel prognosis system that isn’t effected by tumor site.

Tumorigenesis results from the interplay of multiple factors. With a better understanding of the etiology and pathogenesis of CC, as well as treatment strategies such as surgery and chemoradiotherapy, the survival rate of CC has significantly improved. Several limitations to single therapy and prognostic evaluation of CC, however, have contributed to the high mortality rate associated with advanced CC. At John Hopkins University, Le et al. (2015) discovered that mCRC patients with mismatch repair-deficient (dMMR) or microsatellite instability-high (MSI-H) can benefit from immune checkpoint inhibitors (ICIs). Thus, researchers have evaluated immunotherapy for CC (Le et al., 2015). Numerous studies have established that immunotherapy can benefit a significant number of dMMR/MSI-H patients. Microsatellite stability (MSS) tumors, in theory, have less immune cell infiltration and expression of immune-related genes than MSI tumors. Then, as a result of increased immunocyte infiltration, MSI tumors express neoantigen more easily, making them more sensitive to immunotherapy (Lin et al., 2020). Mismatch repair (MMR), on the other hand, can recognize and fix mutation errors, hence preventing the mutant protein from leading to a tumor. Defects in MMR-related genes result in impaired repair function and the accumulation of numerous altered genes throughout the DNA synthesis process. Tumor mutation burden (TMB) is enhanced by DNA impairment, and genome stability is affected to some extent. Previous research has shown that increased TMB may promote the translation of mutant proteins and stimulate the generation of neoantigens via major histocompatibility complex (MHC) binding (Sha et al., 2020). Meanwhile, elevated TMB can improve the immunogenicity of MSI tumors, resulting in a better immunotherapy outcome than before. Previous research also reports that 97% of MSI-H tumors have TMB ≥10 mutations/Mb (Chalmers et al., 2017). Additionally, a portion of MSS tumors contains a high concentration of TMB, which promotes the enrichment of activated CD4 and CD8 T cells, hence enhancing the tumor’s response to ICIs (Ghorani et al., 2020). Numerous studies have demonstrated that TMB can be a reliable predictive index of antitumor response to ICIs (Jiang et al., 2021).

Progress in genomic technology, which began with the completion of the Human Genome Project in 2003, has been accelerated by the advent of transcriptome analysis, biochips (Kozal et al., 1996), and high-throughput sequencing (Reuter et al., 2015). Transcriptome sequencing is currently one of the most widely used high-throughput sequencing technologies, with next-generation sequencing being the most popular (Mosele et al., 2020). Following an integrated analysis of sequencing results, numerous tumor pathological mechanisms have been defined at the molecular level. These findings have aided the development of tumor treatment strategies, with the ultimate goal of translating laboratory findings to the clinic. Currently, the implementation of single transcriptomic analysis has some limitations. For example, there are numerous molecular stages involved in the translation of mRNA to proteins. Certain aberrations in this process may impair protein stability and disrupt the relationship between mRNA and protein levels. The combination of proteomics and transcriptome analysis can accurately uncover the biological mechanisms and clinical transformation of tumors. (Ross et al., 2004) established the concept of quantitative proteomics, a critical component of proteomics research. It is capable of identifying and quantifying all proteins expressed from a single genome or a mixture (Graves and Haystead, 2002). Isotope-based quantitative proteomics has been widely used to analyze specific tumor biomarkers. Numerous isotope-based quantitative proteomics technologies have been developed, including isotope-coded affinity tags (ICATs) (Gygi et al., 1999) and stable isotope labeling of amino acids in cell culture (Ong et al., 2002). Compared with these technologies, isobaric tags for relative and absolute quantification (iTRAQ), developed in 2004 by AB SCIEX, offer significant advantages, including better sensitivity and efficiency (Ross et al., 2004). iTRAQ technology allows for simultaneous labeling of up to eight samples, which may then be analyzed quantitatively using liquid chromatography tandem-mass spectrometry (LC-MS/MS). iTRAQ has been applied to different types of samples, and has yielded significant results, most notably in cancer research. iTRAQ-based studies have identified biomarkers in CRC (Bai et al., 2020), breast cancer (Jézéquel et al., 2019), bladder cancer (Zhang et al., 2017), and other cancers, establishing a solid foundation for further research and analysis of tumor pathogenesis using this technology.

In this study, we first used iTRAQ to analyze six paired samples between the CC and matched samples. After identifying the protein that differed significantly between tumor and normal samples, we obtained mRNA expression data from the TCGA and Gene Expression Omnibus (GEO) databases. To the best of our knowledge, there was rare research that apply differentially expressed protein-related RNA (DEP mRNA) to analyze CC. Surprisingly, there was a significant variation in prognosis and immunocytes infiltration between different DEP mRNA types. The DEP mRNA may then play a unique role in the immunologic microenvironment, influencing whether or not CC patients benefit from immunotherapy. To assess the characteristics of DEP mRNA, we extracted intersecting differentially expressed genes (DEG) from a public database and divided them into three gene clusters. Following that, the DEGs were used to develop a specific system referred to as the DEP-related score (DEPRS), which was used to grade them and compare the high- and low- score groups in terms of prognosis, TMB, MSI status, and immunotherapy sensitivity. This analysis presents a novel and effective strategy for predicting the prognosis of CC and evaluating the therapeutic effects of immunotherapy, without affect by LCC and RCC. Simultaneously, we identified important genes based on DEPRS using weighted gene co-expression network analysis (WGCNA), random forest, and artificial neural networks to better direct clinical work.

## Materials and Methods

**FIGURE 1:**
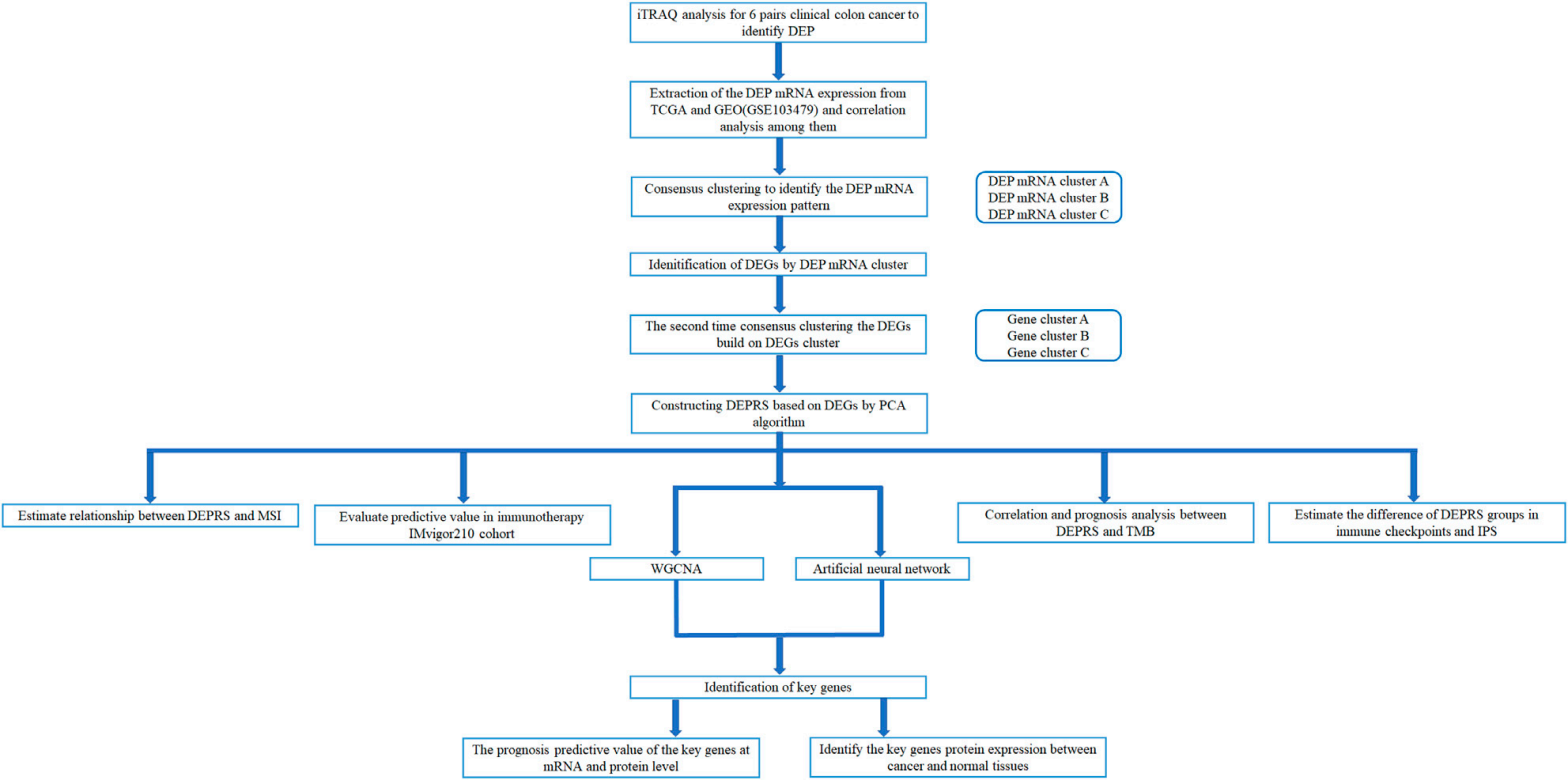
Workflow of whole analysis.

The workflow of this whole study was offered in the Figure 1.

### Collection of Clinical Samples

All human tumor samples and matched adjacent normal samples were collected from patients diagnosed with CC at the Harbin Medical University Cancer Hospital in October 2020. Patients were diagnosed with adenocarcinoma by endoscopy and had not undergone any preoperative chemotherapy or radiotherapy. Tissue samples were immediately stored in liquid nitrogen after resection. This study was approved by the Ethical Committees of Harbin Medical University Cancer Hospital, and patients signed an informed consent form.

### Colon Cancer Transcriptome Data Download and Preprocessing

In this study, we downloaded 629 CC RNA-sequencing data from two high-throughput sequencing platforms; 473 cases from TCGA and 156 cases from GEO (GSE103479) (http://www.ncbi.nlm.nih.gov/geo/). The above data included information about each patient’s somatic mutation, primary tumor site, clinical stage, and survival status. The tumor was characterized as RCC when it was located in the cecum, ascending colon, and hepatic flexure of the colon. The primary location of the tumor was the splenic flexure of the colon, descending colon, sigmoid and recto-sigmoid junction and were defined as LCC. After excluding patients with incomplete survival data, 444 samples were included in the study; TCGA 322 and GEO 122. We obtained the normalized matrix files from GEO for the microarray data. For data from TCGA, we downloaded the RNA-sequencing data (FPKM value) of gene expression, which was then converted into transcripts per kilobase million (TPM) values for combined analysis. To adjust the batch effect caused by non-biotechnology deviation, the “Combat” function of the R package “SVA” was used.

### Unsupervised Clustering Based on Differentially Expressed Protein-Related mRNA

iTRAQ was used to examine six pairs of left- and right- colon cancer and matched normal samples, and 243 DEPs (|log2foldchange| > 0.5, *p*-adj < 0.05) were identified (Detail iTRAQ method and protein information in Supplementary Data). Then, DEPs related mRNA expression levels were extracted from the integrated data set. The DEP mRNA was used to distinguish between tumor types. The hierarchical agglomerative cluster was used to cluster all tumor samples in the R package “ConsensusClusterPlus” (Wilkerson and Hayes, 2010). Cluster count and membership were identified through unsupervised analysis using stability evidence. To ensure the cluster’s stability, the analysis was repeated 1,000 times.

### Gene Set Variation Analysis

To compare the biological processes of DEP mRNA in distinct tumor types, we utilized the R package “GSVA” to perform enrichment difference analysis. GSVA uses a nonparametric and unsupervised method to estimate the variation pathway and enrichment of biological processes across several expression datasets. Following that, we download Gene Ontology (GO) and Kyoto Encyclopedia of Genes and Genomes (KEGG)-related databases from the MSigDB database (http://softwar.broadinstiture.org/gsea/msigdb/) to finish the GSVA analysis. The heat map displays the significantly distinct pathway in the analysis results (*p*-adj < 0.05).

### Evaluation and Difference of Immune Infiltration in Different Types of DEP mRNA

To estimate immunocyte infiltration of samples, the R package “GSVA” was used to analyze single-sample gene-set enrichment analysis (ssGSEA). We acquired information on immune cell marker gene expression from Charoentong’s Charoentong et al., 2017 research and calculated the enrichment coefficients, which showed the relative abundance of immune cells in samples. Finally, we compared immune infiltration patterns across various DEP mRNA clusters.

### Identification of Differentially Expressed Genes Between Differentially Expressed Proteins mRNA Clusters

R package “Limma” was used to identify DEGs (*p*-adj < 0.01) in different DEP mRNA clusters (Ritchie et al., 2015). Following that, numerous sets of DEGs were obtained, and the intersection was used to extract their expression in all samples for subsequence processing. The intersecting DEGs were analyzed using the R package “clusterProfiler” (Yu et al., 2012), which included GO and KEGG functional annotations analysis.

### Construction of Differentially Expressed Proteins Related Scores

We developed an algorithm and defined it as DEPRS, for quantification of DEP mRNA-related types in LCC and RCC. The entire process was as follows: Firstly, the intersecting DEGs were subjected to univariate Cox proportional hazard regression analysis (COX) using the R package “glmnet” (Friedman et al., 2010). The gene that had a significant effect on prognosis was standardized. Based on these DEP mRNA-associated DEGs, the unsupervised clustering method was used to cluster all patients for further analysis. Meanwhile, we performed principal component analysis (PCA) to identify the key components of these genes to construct a DEP mRNA-related gene signature. Principal component 1 and principal component 2 were chosen as the signature scores. Finally, we used an approach comparable to the gene expression level index to calculate each patients’ DEPRS: DEPRS= ∑PCA1i+∑PCA2i (i is the expression of DEP mRNA-related DEGs). The best cut-off value for dividing high and low DEPRS groups for prognosis was obtained using the R package “maxstat” (Laska et al., 2012).

### Prediction of Immunotherapy Sensitivities

Immunotherapy sensitivities were estimated in the high and low DEPRS groups from two perspectives: immune checkpoint-related genes immunophenoscores (IPS) and open-label immunotherapy cohort. Various immune-related genes, including effector cells, immune suppressor cells, MHC molecules, and immunoregulatory cytokines were used to determine immunogenicity. Machine-learning algorithms can accurately estimate and quantify immunogenicity. The IPS of CC in TCGA was downloaded from the TCIA database (https://tcia.at/). Then, we compared the immunophenoscore differences between high and low DEPRS groups in immunotherapy, to predict immunotherapy sensitivities. Simultaneously, a thorough search for gene expression profiles in publicly available immunotherapy cohort of metastatic urothelial tumors (IMvigor210: http://research-pub.gene.com/IMvigor210CoreBiologies) Mariathasan et al., 2018 was performed. Data were pretreated using the R package “IMvigor210CireBiologies.” The RNA-SEQ data was filtered and normalized using the R package “edgeR,” and transformed using voom in the R package “limma.” In addition, we downloaded and organized prognostic status and therapeutic effect data. Based on the above computation, the DEPRS for each sample in this cohort was calculated and divided into high and low score groups to compare the difference in therapeutic response between the two groups.

### Identification of Key Genes

To screen and identify DEP-related prognostic key genes in LCC and RCC, two methods were used: weighted gene co-expression network analysis (WGCNA), random forest, and artificial neural network. First, we used the combination of DEP mRNA and DEPRS to run the WGCNA. To transform the adjacency matrix (AM) to a topological overlap matrix with DEGs, the appropriate power index was selected. The higher the value of the mean connectivity, the more the network conforms to the scale-free characteristics. According to the relationship between soft threshold (power) and mean connectivity, the minimum index when the *R* ^2^ of scale-free network reaches 0.8 was taken as the appropriate index. Then, a correlation between gene consensus modules and DEPRS was established, and gene significance (GS) was defined as the mediated *p*-value of each gene (GS = lgP) in a linear regression between gene expression and the scores. Subsequently, GS >0.6 genes in the module with the highest positive correlation coefficient with DEPRS were screened. Following that, the random Forest software package was used to classify all DEP mRNA. The parameter mtry (Optimal variable number of binary trees in the nodes) was set to 6, and the optimal number of trees included in the random forest was 49. After constructing the random forest module, the dimensional importance value (IV) was determined using the module’s decreasing accuracy method (Gini coefficient method). Genes with a length of more than two were treated as special genes to construct subsequent modules. Unsupervised hierarchical cluster analysis was performed on these specific genes and a heat map was generated to demonstrate their classification effect. Following that, we classified these genes into high and low expression groups based on their median expression and categorized them as gene scores (low expression as 0, high expression as 1). On significant variables, the R package “Neuralnet” was used to construct an artificial neural network model. The model’s hidden layer parameter was set to 5, and the outcomes of weight score multiplied by gene score were used to construct a classification model for HDEPRSG and LDEPRSG. The R package “pROC” was used to create the receiver operating characteristic curve (ROC), as well as calculate the area under the curve to confirm classification performance. Finally, the specific gene was obtained from an intersection of the particular gene and the screened gene. To repeatedly validate the important gene at the protein level, we downloaded the proteomics cohort in the TCGA COAD sample (including 29 normal samples and 64 tumor samples) from The Clinical Proteomic Tumor Analysis Consortium (CPTAC) (https://proteomics.cancer.gov/programs/cptac). Additionally, we investigated the prognostic significance and expression of key genes.

### Statistical Analyses

All the statistical analyses were performed using R-version 4.0.5. The Wilcoxon test and Kruskal-Wallis test were used to compare two groups, and more than two groups respectively. The prognosis curve was constructed using the Kaplan-Meier plotter, and the log-rank test was used to determine whether there was a statistically significant difference in prognosis. Spearman correlation coefficient was used to measure the relationship between variables. The mutation gene status in different groups was demonstrated using the R package “maftool” (Mayakonda et al., 2018). *p* < 0.05 was considered statistically significant.

## Results

### Differentially Expressed Proteins mRNA Landscape in Colon Cancer

**FIGURE 2:**
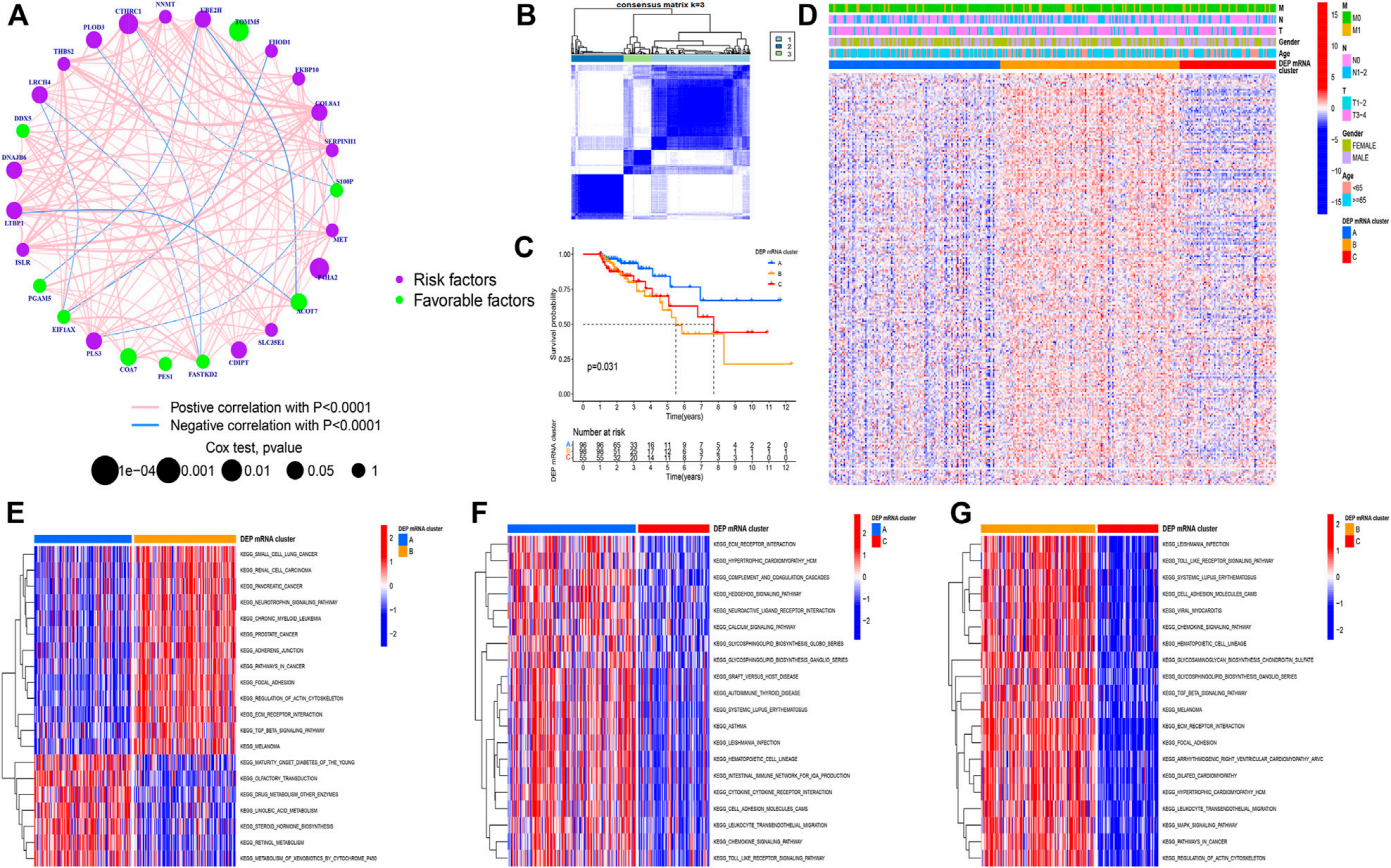
**(A)** Correlation among DEP mRNA in CC. The circle size represents the effect of each regulator on the prognosis. The range of values calculated by Log-rank test was *p* < 0.001, *p* < 0.01, *p* < 0.05, and *p* < 0.1, respectively. The green dots in the circle represent favorable prognostic factors; Purple dots in the circle represent prognostic risk factors. The lines linking regulators indicate interactions, and their thickness shows correlation strength between genes. Negative correlation is marked in blue and positive correlation in red. **(B)** Consensus matrixes of all CC samples for appropriate k value (k = 3), displaying the clustering stability using 1,000 iterations of hierarchical clustering. All samples were clustered into three subtypes. **(C)** Survival analyses for the three DEP mRNA cluster based on 1,051 patients with CC from GEO cohorts including 96 cases in cluster-A, 98 cases in cluster-B, and 55 cases in cluster-C. Kaplan-Meier curves with Log-rank *p*-value = 0.031 showing significant survival difference among the three DEP mRNA patterns. The DEP mRNA cluster A had significantly better overall survival than the other two clusters. **(D)** A heat map showing the unsupervised clustering of DEP mRNA in all CC samples. Columns represent samples. A heatmap visualizing the clinical parameters. Red represents activation and blue represents inhibition. CC cohorts were used as sample annotations. **(E–G)** Results of GSVA enrichment analysis showing different tumor types in the three DEP mRNA patterns. Red represents activation and blue represents inhibition. **(E)** DEP mRNA cluster A vs DEP mRNA cluster B; **(F)** DEP mRNA cluster A vs DEP mRNA cluster C; **(G)** DEP mRNA cluster B vs DEP mRNA cluster C.

The CC samples and matched normal samples were subjected to iTRAQ analysis. Then, 243 DEPs (|log2foldchange| > 0.5, *p*-adj < 0.05) were identified and the corresponding mRNA data were extracted from the integrated dataset. The network was used to depict the comprehensive DEP mRNA landscape. Univariate COX analysis was used to investigate all of the DEP mRNAs, and the interaction between them was demonstrated (Figure 2A). As shown in the figure, there was an explicit negative association between the favorable factor and risk factor. Next, Following that, all CC samples were clustered using the R package “ConsensusClusterPlus” based on 243 DEP mRNA, and 3 clusters were identified (Figure 2B).

A Kaplan-Meier (K-M) curve was used to compare the prognosis of various DEP mRNA clusters. We found that DEP mRNA cluster B had a significantly worse prognosis than the other two clusters (*p* = 0.031) (Figure 2C). Then, DEP mRNA expression in the three clusters was shown using a heat map, and the expression of the 3 clusters was significantly different (Figure 2D). The KEGG-related GSVA was used to investigate the biological function of these DEP mRNA clusters. When cluster A was compared to cluster B, we observed that DEP mRNA cluster B was significantly more abundant in carcinogenic activation pathways, such as small cell lung cancer, renal cell carcinoma, adherens junction, and TGF-β pathway (Figure 2E). Then, GSVA analysis between clusters A and C revealed that cluster A enriched for a variety of pathways not previously associated with it, including ECM receptor interaction, chemokine pathway, and calcium signaling pathway (Figure 2F). In comparison to clustering C, DEP mRNA cluster B enriched for a variety of carcinogenic activation pathways including the MAPK signaling pathway, focal adhesion, etc. (Figure 2G). As a result of the aforementioned finding, DEP mRNA cluster B was found to have the worst prognosis among three clusters and an enrichment analysis with numerous carcinogenic pathways.

### Differentially Expressed Genes Selected from Differentially Expressed Proteins mRNA Clusters

**FIGURE 3:**
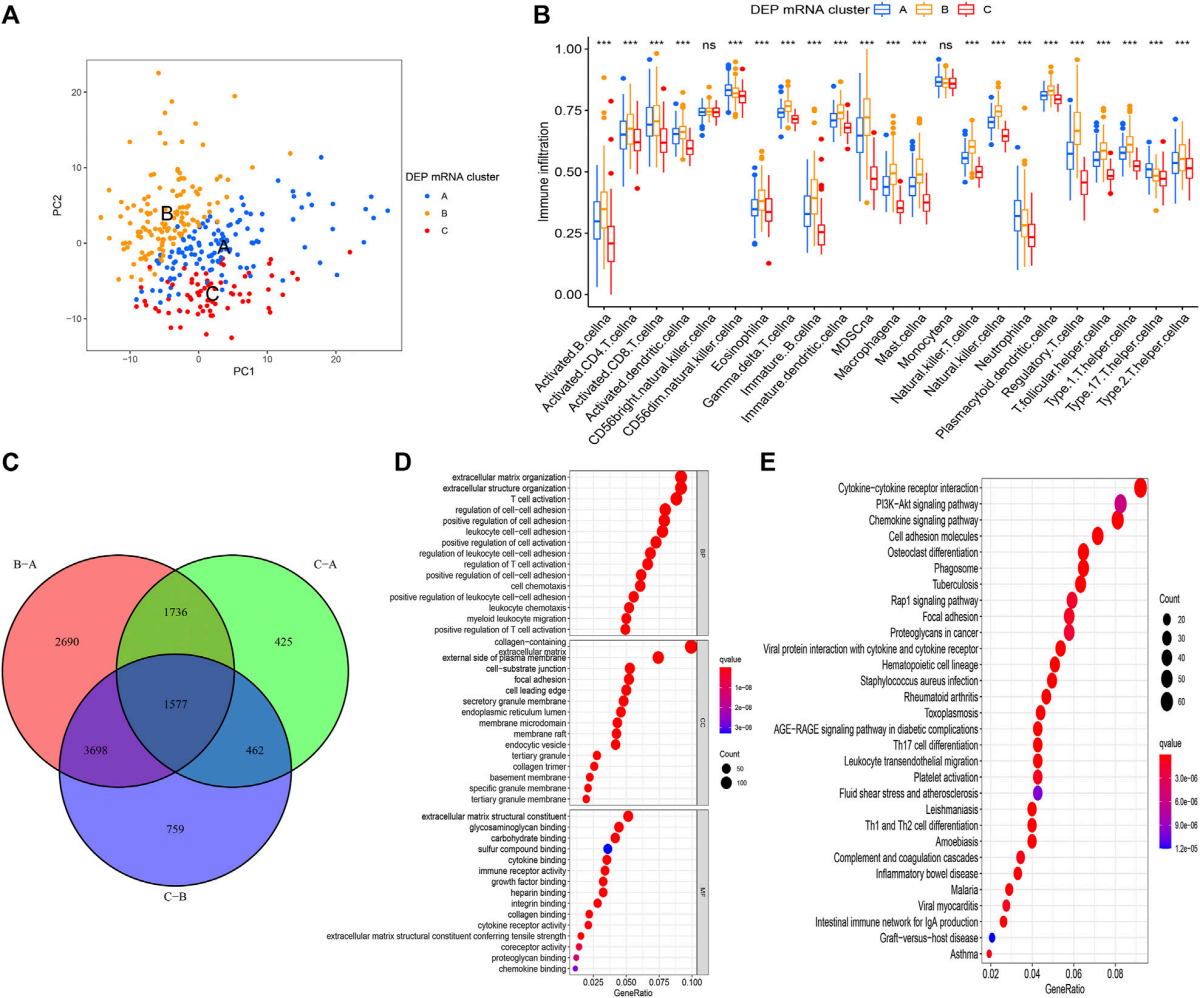
**(A)** PCA for the transcriptome profiles of three DEP mRNA clusters showing significant differences in transcriptome between different clusters. **(B)** The abundance of each TME infiltrating cell in the three DEP mRNA clusters. The upper and lower ends of the boxes represent interquartile range of values. The lines in the boxes represent the median value, and the colored dots represent outliers. The asterisks represent the statistical *p*-value (*p < 0.05; **p < 0.01; ***p < 0.001). **(C)** The venn diagram showing the overlapping genes between the three clusters. **(D)** GO enrichment analysis of the overlapping gene signatures. **(E)** KEGG enrichment analysis of the overlapping gene signatures.

The PAC clustering method was used to confirm the DEP mRNA clustering result previously obtained. As a result of the PCA grouping, three distinct DEP mRNA groups were identified, indicating the consistency and accuracy of the test (Figure 3A). The DEP mRNA cluster B showed a poor prognosis and was associated with the cancer signaling pathway. The ssGSEA was used to evaluate each sample to determine the link between the three clusters and immune infiltration. Surprisingly, an investigation of immunological infiltration revealed that DEP mRNA cluster B was significantly abundant in immune cells such as MDSC, activated B cell, activated CD4 T cell, and natural killer cell (Figure 3B). However, when compared to clusters A and C, cluster B showed no advantage in terms of survival time. To further investigate the differences in the immunological microenvironment of DEP mRNA, the R package “Limma” was used to identify DEG between different DEP mRNA clusters. There were 1,577 overlapping DEGs (Figure 3C). Additionally, using the R package “clusterProfiler” the DEGs were subjected to GO and KEGG analysis. In GO enrichment, the overlapped DEGs were enriched in immune response (Figure 3D), while in KEGG they were enriched in inflammation-related pathways and carcinogenic pathways (Figure 3E).

### Construction of Differentially Expressed Proteins Related Score

**FIGURE 4:**
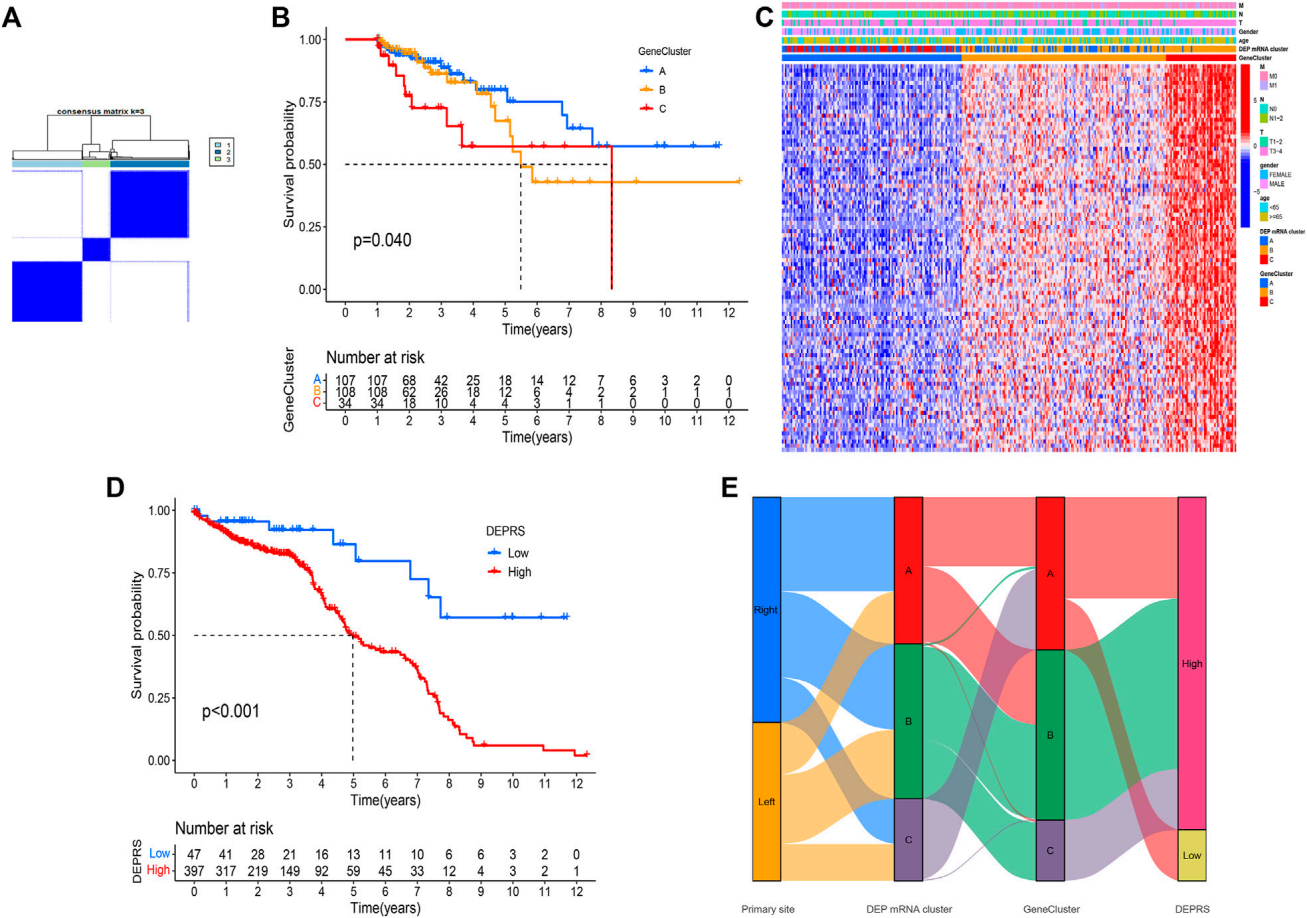
**(A)** Consensus matrixes of TCGA-COAD cohorts for appropriate k value (k = 3), displaying the clustering stability using 1,000 iterations of hierarchical clustering. TCGA samples were clustered into three subtypes based on the DEGs among three DEP mRNA clusters. **(B)** Kaplan–Meier curves showing the overall survival across the gene clusters. The log rank test showed an overall *p* = 0.040. **(C)** A heat map showing the expression of DEGs in different gene clusters. Heat map colors indicate relative DEGs expression levels. **(D)** Kaplan–Meier curves showing the overall survival in high-DEPRS and low-DEPRS groups. The log rank test showed an overall *p* < 0.001. **(E)** The Sankey diagram displaying the distribution of patients with primary tumor sites, DEP mRNA clusters, gene clusters, and DEPRS.

To conduct in-depth research on the various expression types of DEP that contribute to the differences in the immune microenvironment, the TCGA-COAD sample was re-divided into three gene clusters based on overlapping DEGs (Figure 4A). To determine the survival difference between the three gene clusters, a prognostic analysis was performed. Although the results presented on the survival curve weren’t perfect, but the novel gene clusters were shown the significant difference of prognosis among different clusters (*p* = 0.04) (Figure 4B). Following that, the DEGs expression in distinct gene clusters and DEP mRNA clusters was visualized using the heat map (Figure 4C). Taking into account the complexity and variability of individual differential protein expression patterns and the subsequent identification of important genes, we developed a novel algorithm called DEPRS to quantify the DEP mRNA expression in individual patients. The optimal cut-off value was determined using the R package “maxstat,” and patients were divided into high DEPRS and low DEPRS groups (HDEPRSG and LDEPRSG). As shown in the prognostic analysis, LDEPRSG had a better prognosis than HDEPRSG (*p* < 0.001) (Figure 4D). Following that, a Sankey diagram illustrating the distribution of patients with tumor sites, DEP mRNA cluster, gene clusters, and DEPRS was displayed (Figure 4E). As indicated in Figure 4E, CC was divided into LCC and RCC, which were further divided into three DEP mRNA clusters. Following that, the DEP mRNA clusters were stratified into three gene clusters. Surprisingly, samples in the DEP mRNA cluster B and gene cluster B were classified as having a high DEPRS score, indicating a poor prognosis. A portion of DEP mRNA cluster B occupied a section of gene cluster B, but partial samples in DEP mRNA cluster A were retained in gene cluster A distributed in low DEPRS, which was associated with a better prognosis. It was indirectly demonstrated that multiple clustering modes produced consistent results. Following the above result, the difference between LCC and RCC wasn’t distinct that means the DEPRS model own the special advantage to ignore tumor site.

### Differentially Expressed Proteins Related Score and Correlation Analysis of Somatic Mutation

**FIGURE 5:**
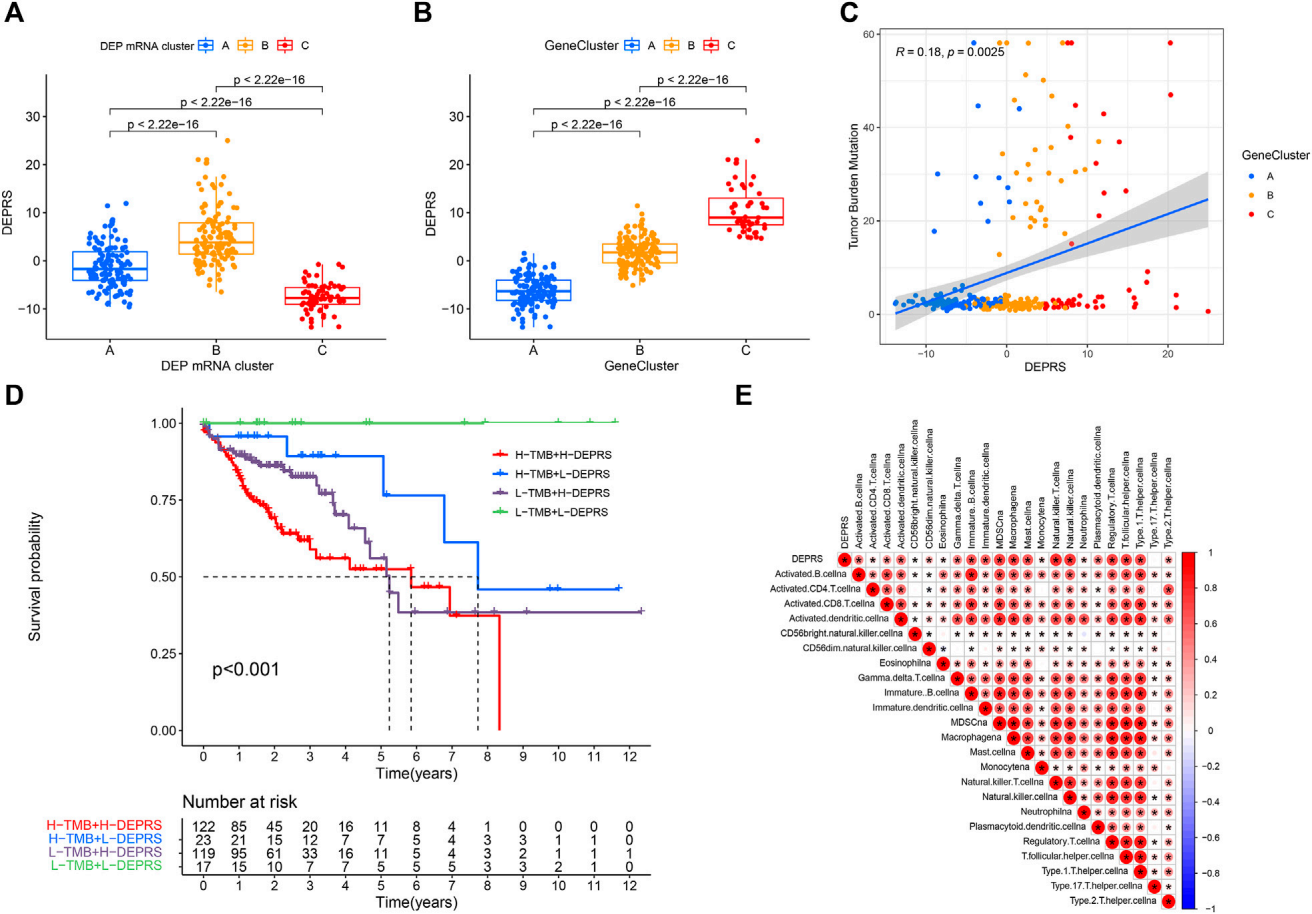
**(A)** Differences in DEPSR among the three DEP mRNA clusters in TCGA cohort. Statistical comparisons were made using Kruskal-Wallis test (*p* < 0.001). **(B)** Differences in DEPSR among three gene clusters in TCGA cohort. Statistical comparisons were made using Kruskal-Wallis test (*p* < 0.001). **(C)** The scatterplots show positive correlation between DEPRS and TMB. The Spearman correlation between DEPRS and TMB was 0.18 (*p* = 0.0025). **(D)** Kaplan–Meier curves showing the overall survival based on TMB and DEPRS. Log rank test, *p* < 0.001. **(E)** Correlations between DEPRS and the universal landscape of immune cell interaction in TME as determined using Spearman analysis. Negative correlation is shown in blue and positive correlation is shown in red.

**FIGURE 6:**
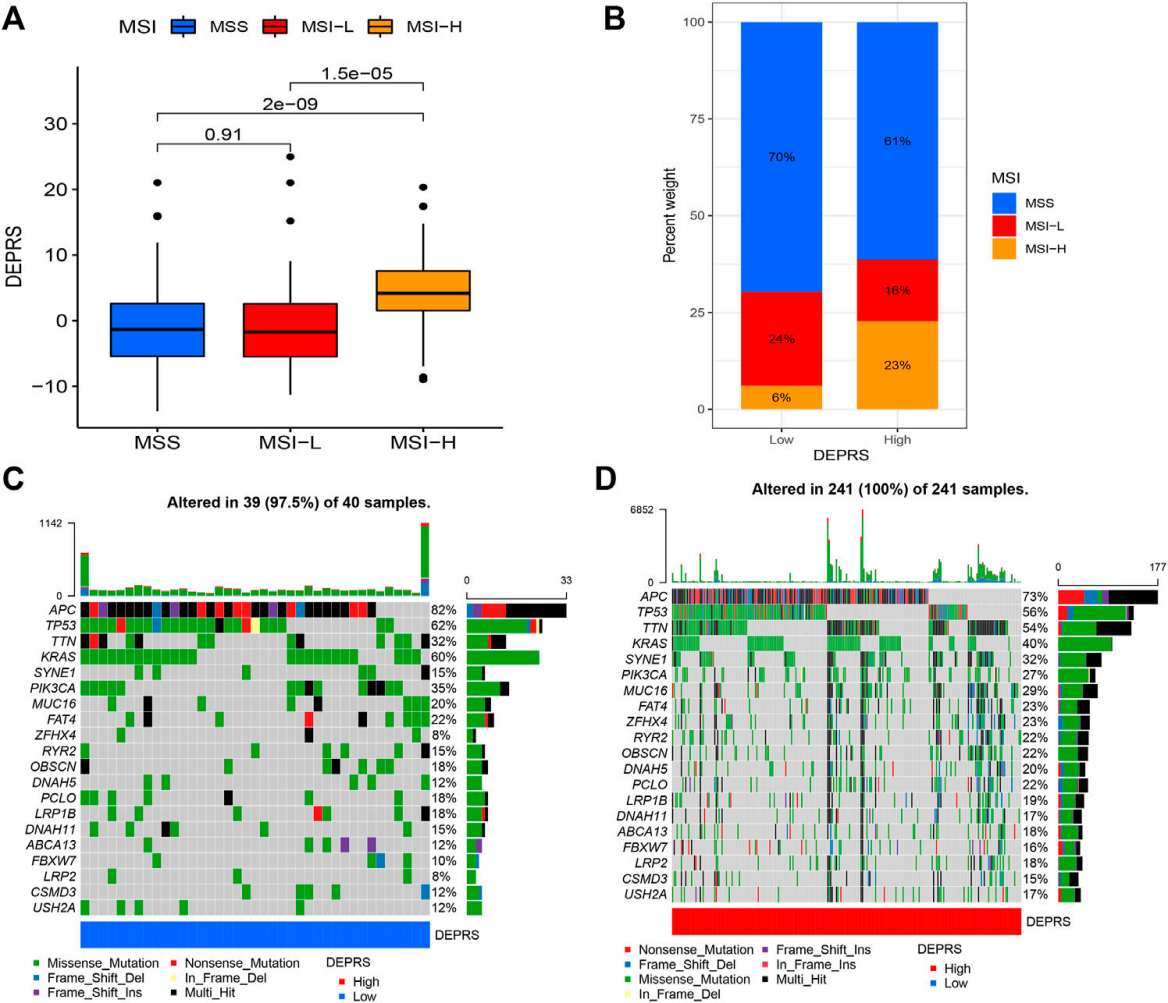
**(A)** The profile of DEPSR among different MSI status. The Kruskal-Wallis test was adopted to make statistical comparisons between different MSI status (*p* < 0.001). **(B)** Proportion of patients with different MSI status in high DEPES and low DEPRS groups. The proportion of MSS and MSI-L patients in the low DEPRS group was significantly lower than that in the high DEPRS (*p* < 0.05). The groups were compared using the Kruskal-Wallis test. **(C,D)** A waterfall diagram showing the top 20 driver genes with the highest mutation frequency in low DEPRS **(C)** and high DEPRS **(D)** groups.

To further illustrate the relationship between DEPRS and the preceding two cluster modes, we examined the correlation between the obtained clusters and DEPRS. There was a significant difference in DEPRS between various DEP mRNA clusters (Figure 5A). The DEP mRNA cluster B had had a much higher median DEPRES value than the other two clusters. Then, when compared to other clusters, gene cluster C had significantly increased DEPRS (Figure 5B). Because the DEG was shown to be enriched in immune-related pathways, we examined the correlation between TMB and DEPES. There was a significant correlation between TMB and DEPRS using association analysis (Coefficient: R = 0.018, *p* = 0.0025) (Figure 5C). With an increase in DEPRS, the distribution of gene clusters followed a significant rule. TMB showed an increasing tendency as DEPRS increased, indicating that patients with high TMB have a poor prognosis. According to the preceding data, gene cluster A had a low DEPRS and a low TMB, indicating a better prognosis. Additionally, we examined the effect of TMB and DEPRS integration on prognosis. All samples were stratified into high- and low- TMB subgroups. K-M curves were used to analyze the combined effect of TMB and DEPRS on prognosis. Within the same DEPRS status, the group with a high TMB had a worse outcome than the group with a low TMB. Nonetheless, TMB status did not affect the DEPRS predictive ability of prognosis, such that patients with high DEPRS invariably had a poor prognosis (Figure 5D). We also created a correlation map to visualize the DEPRS and immune cell interaction in TME, owing to the relationship between TMB and DEPRS. It is demonstrated unequivocally that the characteristics of DEPRS were highly correlated with high levels of immunocyte infiltration (Figure 5E). We aimed to determine the association between MSI status and DEPRS because it was critical for immunotherapy sensitivity. MSI-H differed significantly from MSS and MSI-L in this study, indicating that MSI-H was associated with a high DEPRS (Figure 6A). Consistent with this finding, 23% of MSI-H patients had high DEPRS compared with 6% with low DEPRS (Figure 6B). In addition, we compared the differences in somatic variation driver genes between individuals with high and low DEPRS. The top 20 driver genes exhibiting the highest mutation frequency were selected (Figures 6C,D). The mutation rate of the majority of driver genes was higher in the HDEPRSG than in the LDEPRSG.

### Assessment of Differentially Expressed Proteins Related Score in Predicting Immunotherapy Efficacy

**FIGURE 7:**
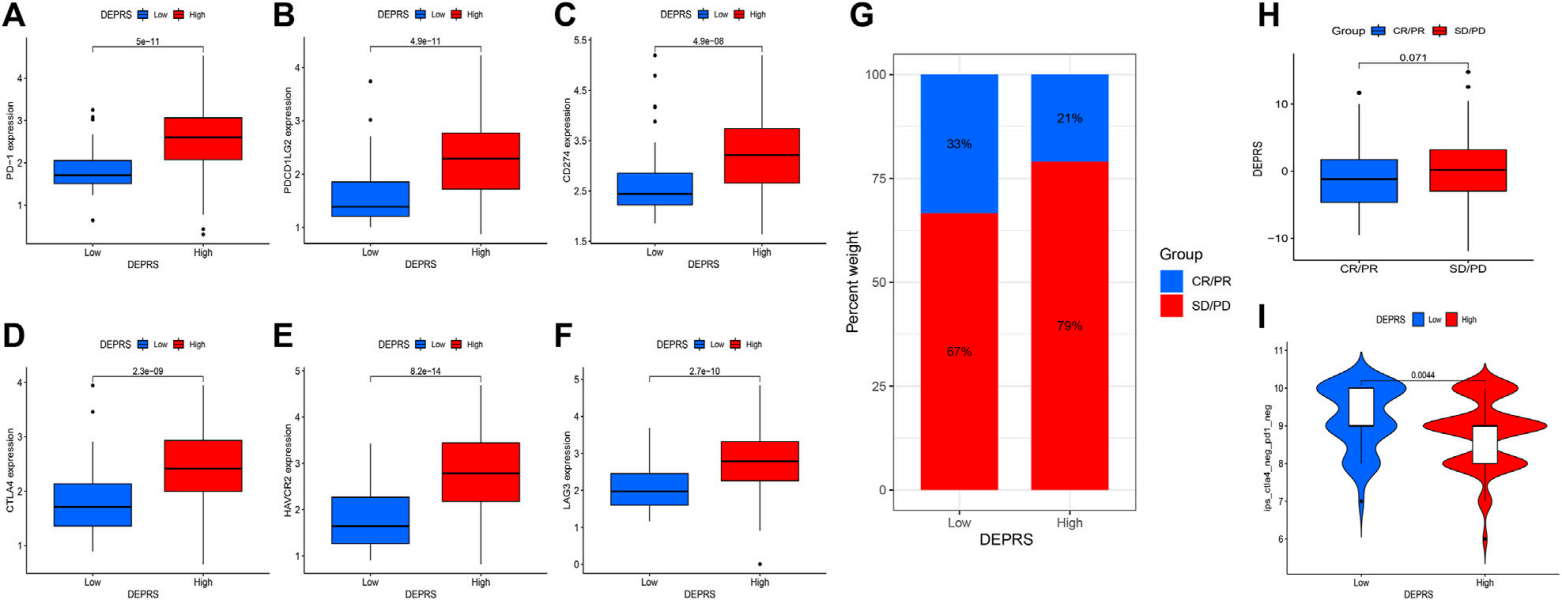
**(A–F)** Comparison of immunosuppressive checkpoints expression between high DEPRS expression and low DEPRS expression groups. The expression of PD-1 **(A)**, PDCD1LG2 **(B)**, CD274 **(C)**, CTLA4 **(D)**, HAVCR2 **(E)** and LAG3 **(F)** was higher in high DEPRS than in the low DEPRS expression group (all *p* < 0.05). Statistical comparisons were done using the Wilcoxon test. **(G)** Proportion of patients with different treatment outcomes in high DEPRS and low DEPRS. The proportion of CR/PR patients in high DEPRS was lower than that in low DEPRS (21 vs 33%). **(H)** Comparison of DEPRS between different treatment outcome groups (*p* = 0.071). **(I)** The relationship between IPS and DEPRS groups in patients (*p* = 0.0044).

The purpose of this study was to compare immunotherapy sensitivity parameters in two DEPRS groups. For immune checkpoints, PD-1, PDCD1LG2, CD274, CTLA4, HAVCR2, and LAG3 were significantly expressed in HDEPRSG than LDEPRSG (*p* < 0.05) (Figures 7A–F). Therefore, the parameters indicated that different groups responded differently to immunotherapy. Additionally, we demonstrated the stability of DEPRS as a predictor of immunotherapeutic efficacy in a public immunotherapy cohort. The relationship between DEPRS and treatment outcome was shown in Figure 7G. The results showed that the stable disease (SD)/progression disease (PD) proportion was significantly higher in the high DEPRS than in the low DEPRS group (79 vs 67%) and that SD/PD patients had a higher DEPRS than the complete response (CR)/partial response (PR) group (*p* =0.071) (Figure 7H). On the other hand, the relationship between IPS and DEPRS was used to estimate the predictive potential of DEPRS. The IPS, which measures immunogenicity, was a significant difference between DEPRS groups, with the IPS in the high DEPRS group being lower than the IPS in the low DEPRS (*p* = 0.0044) (Figure 7I). Based on these findings, we hypothesized that DEPRS may possess the ability to predict prognosis and immunotherapy efficacy.

### Identification of Key Genes in Differentially Expressed Proteins Related Score

**FIGURE 8:**
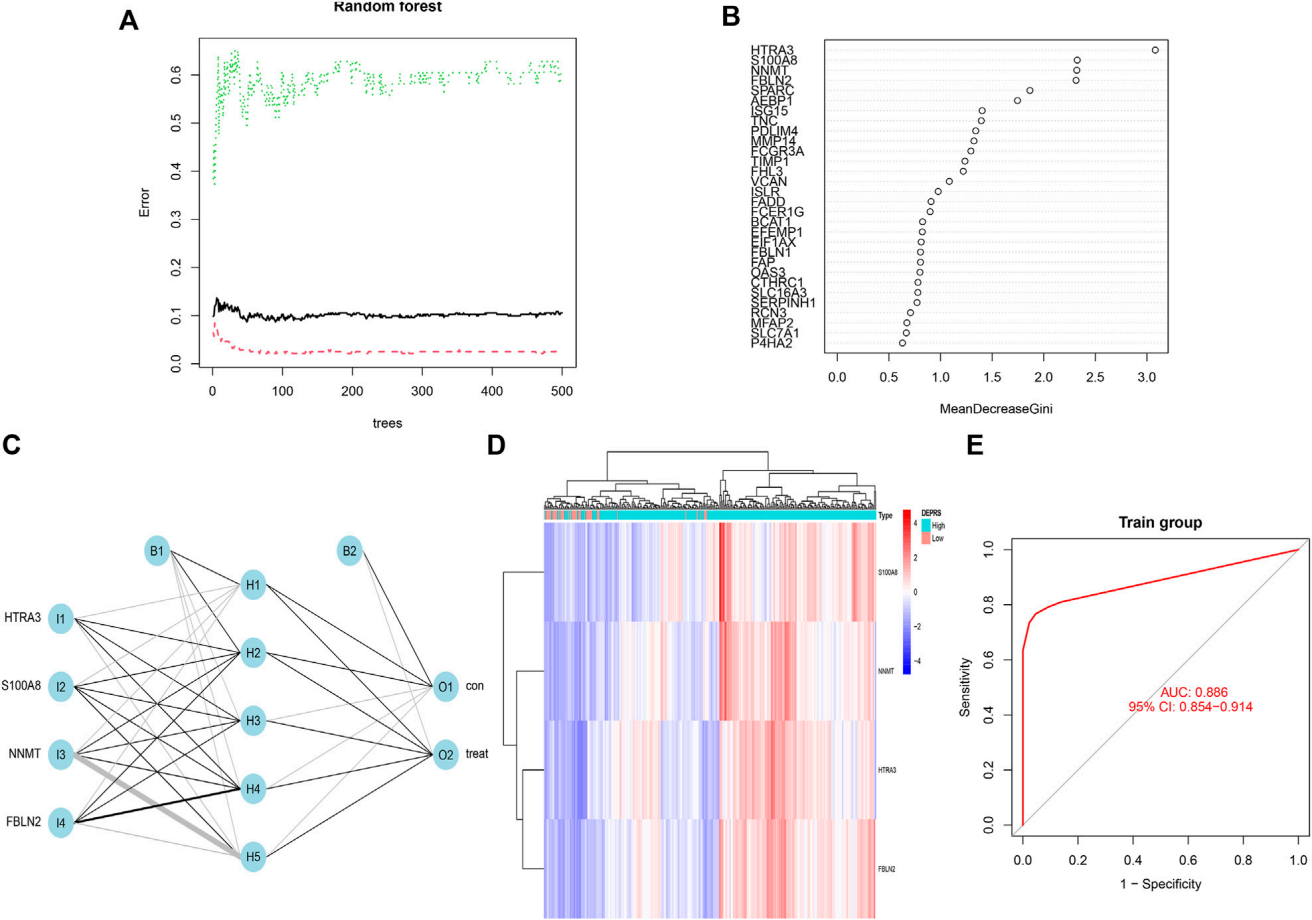
**(A)** Relationship between the error rate and number of classification trees. **(B)** Gini coefficient method used to screen specific genes (IV >2). There were HTRA3, S100A8, NNMT, FBLN2. **(C)** Results of neural network visualization. **(D)** A heat map showing differences among the four genes between high DEPRS and low DEPES. Red color indicates positive expression and blue indicates negative expression. **(E)** The predictive value of the artificial neural network model based on four key genes in immunotherapy efficacy (AUC, 0.886, 95% CI: 0.854–0.914).

**FIGURE 9:**
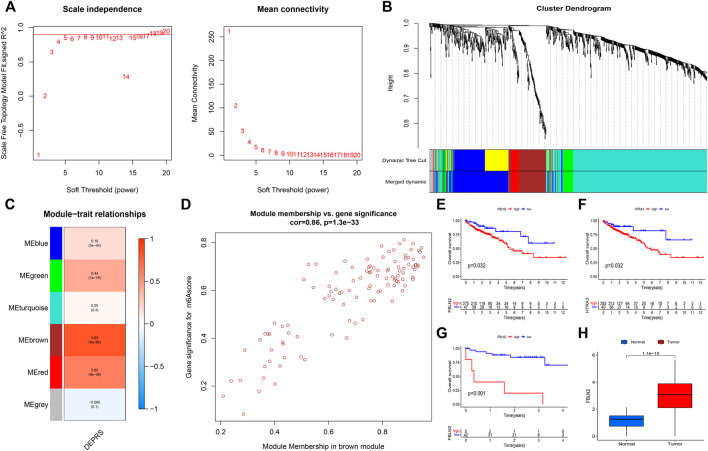
**(A)** To achieve a scale-free co-expression network, the power index = 5 was chosen as the appropriate soft threshold. **(B)** The branches of the dendrogram correspond to six different gene modules. **(C)** The correlation between gene modules and DEPRS. Each cell contains corresponding correlation coefficient and *p*-value. **(D)** Significant positive correlation between module membership and gene significance (Correlation coefficient = 0.86, *p* < 0.001). **(E,F)** Kaplan-Meier curves showing the overall survival of the two key genes FBLN2 (*p* = 0.032) **(E)** and HTRA3 (*p* = 0.032) **(F)**. Red represents high expression, and blue represents low expression. **(G)** The difference in OS between high and low FBLN2 expression groups (*p* < 0.001). **(H)** The difference in FBLN2 expression between cancer tissues and normal tissues at protein level.

The random forest module was constructed to identify key genes based on DEPRS, and Figure 8A depicts the relationship between reference model error and the number of decision trees (Figure 8A). A total of 49 decision trees were selected since the error rate was the lowest and the most relatively stable. The IV > 2 genes (HTRA3, S100A8, NNMT, and FBLN2) were chosen for further analysis using the Gini coefficient method (Figure 8B). Unsupervised hierarchical cluster analysis was used to study these specific genes, and the resulting heat map was sued to illustrate the relationship between their expression and DEPRS status (Figure 8C). The expression of these four genes was significantly increased in the high DEPRS group. Next, these four genes were used to construct a neural network prediction model as illustrated in Figure 8D. The ROC curve was used to determine the sensitivity of the module, and the AUC value was 0.886 (Figure 8E). The results suggested that the neural network prediction model based on four specific key genes produced satisfactory results and that HDEPRSG was more accurately classified than LDEPRSG. On the other hand, a gene co-expression network based on DEPRS was constructed to identify the key DEP mRNA. By selecting number 5 as the appropriate soft threshold (Figure 9A), a scale-free co-expression network was constructed (Figure 9B), yielding six modules. The brown module had the highest correlation with DEPRA (coefficient = 0.83, *p* < 0.001) (Figure 9C). After interacting with the four special genes identified by the neural network, two key genes HTRA3 and FBLN2 were obtained. The K-M curves were used to demonstrate that the high expression of two genes was associated with a poor prognosis (Figures 9E,F). At the protein level, high FBLN2 expression was associated with a poor prognosis (*p* < 0.001) (Figure 9G), and FBLN2 expression was significantly higher in tumor samples than in normal samples (*p* < 0.001) (Figure 9H).

## Discussion

Numerous studies have analyzed single transcriptome, resulting in a lack of stability for estimating prognosis and immunotherapy. In comparison, multi-omics integration research has several distinct advantages in the field of tumor research. To the best of our knowledge, integration of proteomics and transcriptomics research is rare in CC. In this study, iTRAQ analysis was used to identify DEPs, and then extract DEP mRNA expression from a publicly available database. The construction of the DEP mRNA clusters and gene clusters confirmed that various clusters had significant differences. Then, DEPRS were further established to confirm the stability of prognosis prediction and immunotherapy sensitivity for DEP mRNA-related DEGs and its evaluation ability didn’t be affected by LCC and RCC. Finally, key genes that may offer potential clinical value for immunotherapy were identified.

Currently, immunotherapy is considered a novel treatment for cancer. Excepted PD-1, more and more immunotherapy markers were discovered and TMB is an emerging one. TMB refers to the total number of somatic mutations in the tumor genome that it can be used to show the ability of nonsynonymous mutation. It also indirectly reflect the function of tumor producing neoantigen. High levels of neoantigen can easily be recognized by the autoimmune system, and lead to stimulation of CD8^+^ T cells to trigger immune response (Schumacher and Schreiber, 2015). Studies have shown that tumors with high TMB have high number of natural killer (NK) cells and T cells in their tumor microenvironment which indirectly show patients may receive good efficacy of immunotherapy. However, using TMB alone to evaluate the curative effect of immunotherapy is not highly effective. Therefore, researchers have attempted to find other indicators that can be integrated with TMB for the assessment of immunotherapy efficacy. In clear cell renal cell carcinoma, TNFSF14 was highly expressed in the high-TMB group, and the copy number of TNFSF14 was significantly correlated with classical immunocyte infiltration (Xu et al., 2020). A previous study showed that the mutation of ZFHX3 was significantly associated with high TMB and neoantigen load. In addition, the mutation of ZFHX3 showed a strong relationship with high-level T cell infiltration and immune-related genes (Zhang et al., 2021). In cutaneous melanoma, high TMB reflects good prognosis and low grade pathology, increased macrophage M1 and M2, and decreased ratio of Treg cell to memory B cells (Kang et al., 2020). In head and neck squamous cell carcinoma, low TMB level indicates better prognosis than high TMB level, and high immunocyte infiltration (Zhang et al., 2020). In a clinical trial in which patients with advanced melanoma received ipilimumab or tremilimumab treatment, patients in high TMB group (>100 nonsynonymous coding mutations) had longer overall survival (OS) (Snyder et al., 2014). A similar finding was obtained in a Chinese clinical trial on advanced gastric cancer. They found that TMB-H patients showed strong response to toripalimab, and patients who showed double positive TMB-H and PD-had better immune response and improved survival rate (Wang et al., 2019). The integration of TMB and a single gene may be an effective strategy for evaluating immune response to immunotherapy. In our study, we integrated integration proteome and transcriptome data to develop DERPS which can combined with TMB to facilitate evaluation of the prognosis of patients. When patients kept the same level of DEPRS, the high TMB level may remind patients own a bad outcome (Figure 5D).

As shown, immunotherapy effectively controlled MSI-H tumors, and TMB served as an important indicator of immunotherapy efficacy in MSI-H patients. The best cut-off point range of TMB was 37–41 mutations/Mb in MSI-H CRC patients (Schrock et al., 2019). TMB not only can be used as an auxiliary reference index for MSI tumor response to immunotherapy, but also as an indicator of MSI status. Study indicates that tumors lacking the mismatch repair protein duo MLH1/PMS2 always have a lower TMB than those tumor lacking a different protein heterodimer, MLH2/MSH6. Then, even tumor loss the same mismatch repair protein, the different origin of tumor may affect the TMB level (Salem et al., 2020). In our study, MSI-H tumors showed high DEPRS expression (Figures 6A,B), and expression of PD-1, CD174, CTLA4 and other parameter also shown high level in high DEPRS (Figures 7A–F), but tumors with high DEPRS expression had poor response to immunotherapy (Figure 7G). Borrowing this novel score system, we innovatively combined the two parameters to estimate response to immunotherapy. Previous studies mainly emphasize the heterogeneity between LCC and RCC, but the score system own its stability to assess prognosis and efficacy of immunotherapy which didn’t affected by tumor site (Figure 4E). On one hand, further studies are needed to explore the detailed mechanism of TMB and MSI status in CC. On the other hand, our next step try to look for similar gene characters in significant different LCC and RCC, in order to offer accurate evaluation for patients.

To identify the key genes influencing the prognosis and response to immunotherapy, multiple algorithms were applied to screen genes based on DEPRS and DEP mRNA. Two key genes, Fibulin 2 (FBLN2) and HtrA serine peptidase 3 (HTRA3) were identified. HTRA3 was found to be a trimeric protein belonging to the homo-oligomeric serine proteases family. Functionally, HTRA3 was found to play an important role in mitochondrial homeostasis, cell death, and signal transduction (Clausen et al., 2002). A previous study reported that HTRA3 is a pro-apoptotic protein which also suppresses tumor formation. For example, in non-small cell lung cancer, over-expression of HTRA3 inhibited TGF-β1 to suppress tumor metastasis (Zhao et al., 2019). However, persistent expression of HTRA3 results in poor prognosis of CRC. Indeed, high expression of HTRA3 in CRC tumor stroma was associated with adverse outcomes such as high tumor budding (Forse et al., 2017). Evidence from studies has shown that high expression of HTRA3 was correlated with poor prognosis in oral squamous cell carcinoma (Moriya et al., 2015). In this study, we identified another key gene, *FBLN2*. This gene encodes fibulins which is a protein belonging to the extracellular matrix (ECM) glycoprotein family. All FBLN family protein contain epidermal growth factor (EGF)-like domains and a C-terminal structure. Bases on this protein structure, they interact with other proteins to execute their functions (Gallagher et al., 2005). Many studies have shown that FBLN2 can bind to many ligands and function as a scaffold protein in the ECM (Yi et al., 2007). Given that FBLN2 functions in the ECM, downregulation of FBLN2 can promote the migration and invasion of tumor cells thereby causing damage to the basement membrane (Klingen et al., 2021). Furthermore, being a secretory metalloproteinase, ADAMTS-12 participates in tissue remodeling and cell migration. It interacts with FBLN2 to suppress the invasiveness of breast cancer cells. Interestingly, ADAMTS-12 was found to promote tumor development in breast cancer cells lacking FBLN2 by regulating metalloproteinase (Fontanil et al., 2014). In contrast, another study found that FBLN2 promoted tumor growth by interacting with activated β integrin receptor in CRC (Vaes et al., 2021). Consistent with the above finding, high expression level of these two genes (*FBLN2* and *HTRA3*) was linked to worse prognosis in colon cancer (Figures 9E,F), and FBLN2 was found to be significantly differentially expressed in CC (Figure 9H). Further research is needed to clarify their roles during the development of CC.

In summary, this study integrated omics tools ranging from proteomics to transcriptomics to estimate the prognosis and response to immunotherapy. HART3 and FBLN2 were found to be the key genes that can offer predictive role for immunotherapy in CC. Our study provides a reliable method for establishing a quantitative model that can be adopted to explore the pathogenesis of CC. This model may offer its own value to help clinical practice and not affected by tumor site. Then, the application of the model in clinical practice requires further investigation.

## Data Availability

The authors acknowledge that the data presented in this study must be deposited and made publicly available in an acceptable repository, prior to publication. Frontiers cannot accept an article that does not adhere to our open data policies.
