# Supplementary material for: A Novel Quantification System Combining iTRAQ Technology and Multi-Omics Assessment to Predict Prognosis and Immunotherapy Efficacy in Colon Cancer
Source: Front Bioeng Biotechnol. 2022 Apr 4;10:862619. doi: 10.3389/fbioe.2022.862619 (PMC9014007; doi:10.3389/fbioe.2022.862619)
Supplement: Supplementary file 1 [file DataSheet2.docx]

**METHOD**

**1 iTRAQ and LC-MS/MS**

The frozen tumor and normal samples were first ground to powder in liquid nitrogen and incubated in lysis buffer. After protein extraction, relevant reagents were added to the samples to induce reductive alkylation. Next, the concentration of the treated protein was quantitatively assessed with the Bradford method. After the protein concentration was achieved to meet the needs of subsequent experiments, trypsin was added to the EP tube for protein digestion. The obtained peptides were dried and frozen in a vacuum. The dried peptides were dissolved, mixed with equal amounts of iTRAQ reagent, and labeled with iTRAQ reagent for detection. Next, the labeled samples were divided into 12 fractions to complete liquid chromatography coupled with tandem mass spectrometry (LC-MS/MS) via a programmed process.

**2 Sample preparation**

Tissue and normal samples were first ground to powder in liquid nitrogen and incubated in Lysis buffer (7 M Urea, 2 M Thiourea, 4% SDS, 40 mM Tris-HCl, pH 8.5) containing 1 mM PMSF and 2 mM EDTA (final concentration) for 5 min, then 10 mM DTT (final concentration) were added to the sample. The suspension was sonicated for 15 min on ice and then centrifuged at 4 °C, 13,000 rpm for 20 min. The supernatant was mixed with 4 volumes

Of precooled acetone at -20 °C overnight. After centrifugation, the protein pellets were air-dried and resuspended in 8 M urea/100 mM TEAB (pH 8.0). Protein samples were reduced with 10 mM DTT at 56 °C for 30 min, alkylated with 50 mM iodoacetamide (IAM) at room temperature for 30 min in the dark.

After diluted 4 times with 10 mM TEAB, total protein concentration was measured using the Bradford method. Equal amount of proteins from each sample were used for tryptic digestion. Trypsin was added at an enzyme-protein ratio of 1:50, and the digest reaction was performed at 37 ° C for 12-16 hours. After digestion, peptides were desalted using C18 columns and the desalted peptides were dried with Vacuum concentration meter. The dried peptides power was redissolved as 20 μl with 0.5 M TEAB for peptides labeling.

**3 iTRAQ Labeling and Fractionation**

Samples were labeled with iTRAQ Reagent-8 plex Multiplex Kit (AB Sciex U.K. Limited) according to the manufacturer’s instructions. Samples were iTRAQ labelled as following: 1T/4T,115; 1N/4N, 116; 2T/5T, 117; 2N/5N, 118; 3T/6T, 119; 3N/6N, 121. All of the labeled samples were mixed with equal amount. Next, the labeled samples were fractionated using high-performance liquid chromatography (HPLC) system (Thermo DINOEX Ultimate 3000 BioRS) using a Durashell C18 (5μm，100 Å，4.6×250 mm) at high pH conditions. At last, collected fractions were combined into 12 fraction.

**4 LC-MS/MS Analysis**

All protein samples were analyzed using Q Exactive plus mass spectrometer (Thermo Fisher) coupled with the UltiMate 3000 RSLC nano system (Thermo Fisher). Peptides were injected onto a home-made C18 trap column (3μm, 120 Å , 100μm×20mm), and eluted at 300 nL/min onto a C18 analytical column (2μm, 120 Å, 750 μm × 150mm) with a 60min gradient. A binary mobile phase system of buffer A (2% acetonitrile/0.1% formic acid/98% H2O) and buffer B (98% acetonitrile/0.1% formic acid/2% H2O) was used. The LC was interfaced to a Q-Exactive plus quadrupole Orbitrap mass spectrometer via nano-electrospray ionization using an Easy Spray source with an integrated column heater set at 50◦C. An electrospray voltage of 2.2 kV was applied. The mass spectrometer was programmed to acquire, by data-dependent acquisition, tandem mass spectra from the top 20 ions in the full scan from 350 to 1800 m/z. Dynamic exclusion was set to 35s, singly-charged ions 2 were excluded, isolation width was set to 1.6 Da, full MS resolution to 70,000 and MS/MS resolution to 17,500. Normalized collision energy was set to 28, automatic gain control to 2e5, max fill of MS to 20 ms, max fill MS/MS to 100 ms.

**5** **Database Search and analysis**

The resulting MS/MS data were processed using Maxquant (1) (1.6.15.0) search engine (Table 1). The main parameters were set as follows.

Table1.The iTRAQ Databases research and analysis

| Item | Value |
| --- | --- |
| Type of search | iTRAQ 8 plex (Peptide Labeled) |
| Enzyme | Trypsin |
| Fixed modifications | Carbamidomethyl (C) |
| Modifications included in protein quantification | Oxidation (M); Acetyl (Protein N-term) |
| Protein FDR | 0.01 |
| PSM FDR | 0.01 |
| Include contaminants | TRUE |
| Decoy mode | Revert |
| Peptides used for protein quantification | Razor |
| Database | PR1-20090042-uniprot-taxonomy_Homo_sapiens-reviewed_200601.fasta |

1. Tyanova S, Temu T, Cox J. The MaxQuant computational platform for mass spectrometry-based shotgun proteomics. Nature protocols 2016;11:2301-19.
